# Endotrophin neutralization through targeted antibody treatment protects from renal fibrosis in a podocyte ablation model

**DOI:** 10.1016/j.molmet.2023.101680

**Published:** 2023-01-22

**Authors:** Yu A. An, Wei Xiong, Shiuhwei Chen, Dawei Bu, Joseph M. Rutkowski, Joel P. Berger, Christine M. Kusminski, Ningyan Zhang, Zhiqiang An, Philipp E. Scherer

**Affiliations:** 1 Touchstone Diabetes Center, The University of Texas Southwestern Medical Center, Dallas, TX, USA; 2 Department of Anesthesiology, Critical Care and Pain Medicine, McGovern Medical School, The University of Texas Health Science Center at Houston, Houston, TX, USA; 3 Texas Therapeutics Institute, The Brown Foundation Institute of Molecular Medicine, The University of Texas Health Science Center at Houston, Houston, TX, USA; 4 Division of Lymphatic Biology, Department of Medical Physiology, Texas A&M University School of Medicine, Bryan, TX, USA; 5 JP Berger Consulting, 580 Washington Street, #15C, Boston, MA, USA; 6 Department of Cell Biology, The University of Texas Southwestern Medical Center, Dallas, TX, USA

**Keywords:** Endotrophin, Chronic kidney disease, Renal fibrosis, Antibody treatment, Podocytes, Podocin

## Abstract

**Objective:**

Renal fibrosis is a hallmark for chronic kidney disease (CKD), and often leads to end stage renal disease (ESRD). However, limited interventions are available clinically to ameliorate or reverse renal fibrosis.

**Methods:**

Herein, we evaluated whether blockade of endotrophin through neutralizing antibodies protects from renal fibrosis in the podocyte insult model (the “POD-ATTAC” mouse). We determined the therapeutic effects of endotrophin targeted antibody through assessing renal function, renal inflammation and fibrosis at histological and transcriptional levels, and podocyte regeneration.

**Results:**

We demonstrated that neutralizing endotrophin antibody treatment significantly ameliorates renal fibrosis at the transcriptional, morphological, and functional levels. In the antibody treatment group, expression of pro-inflammatory and pro-fibrotic genes was significantly reduced, normal renal structures were restored, collagen deposition was decreased, and proteinuria and renal function were improved. We further performed a lineage tracing study confirming that podocytes regenerate as *de novo* podocytes upon injury and loss, and blockade of endotrophin efficiently enhances podocyte-specific marker expressions.

**Conclusion:**

Combined, we provide pre-clinical evidence supporting neutralizing endotrophin as a promising therapy for intervening with renal fibrosis in CKD, and potentially in other chronic fibro-inflammatory diseases.

## Highlights

• *In vivo* endotrophin neutralization protects renal function post podocyte ablation.
• Endotrophin neutralization ameliorates podocyte ablation resulted inflammation.
• Endotrophin neutralization improves renal fibrosis.
• Endotrophin neutralization potentially promotes podocyte regeneration.
• Neutralizing endotrophin is a promising therapy for renal fibrosis in CKD.

## 1 Introduction

Chronic kidney disease (CKD) affects approximately 10% of the population worldwide [1], and CKD is commonly induced by aging, obesity, diabetes, and glomerulonephritis [2]. Although various factors can contribute to the progression of CKD, both podocyte injury and loss stand out as the main drivers and constitute a very early event for CKD [3,4]. Podocytes are highly differentiated cells that are the major components for the renal filtration barrier. However, podocytes are post-mitotic cells and therefore extremely vulnerable to stress conditions. Upon injury, a maladaptive glomerular response [5,6], including immune activation and the disruption of podocyte cell–cell crosstalk leads to the detachment of podocytes, proteinuria, and eventually culminates in glomerulosclerosis, a major form of renal fibrosis [7]. Recent studies have shown that podocytes have the ability to regenerate after injury and loss [8]. This regeneration process can be modulated. However, there are no effective clinical interventions that promote podocyte regeneration and that result in reduced renal fibrosis and an amelioration of CKD.

The “POD-ATTAC” (podocyte-apoptosis through targeted activation of caspase-8) mouse is a useful model that we have previously described to study podocyte injury and loss [9]. This model takes advantage of inducible podocyte apoptosis, leading to a well-controlled podocyte loss. This model is titratable through the injection of various dosages of the dimerizer (AP20187) that leads to the activation of caspase 8, specifically in podocytes. In our previous studies, we demonstrated that POD-ATTAC mice serve as a good preclinical model of human Focal Segmental Glomerulosclerosis (FSGS) seen in some pathological changes of the kidney. More importantly, at moderate dimerizer dosage, POD-ATTAC mice can restore podocyte density with fully recovered renal filtration function. Therefore, the POD-ATTAC mouse is an ideal system to assess interventions that potentially enhance podocyte regeneration, ameliorate renal fibrosis, and improve renal function.

Endotrophin is a cleavage product of the α3 chain of collagen VI [10]. Since its first identification as a tumor promoting factor [11,12], more and more observations have linked endotrophin to various fibro-inflammatory conditions, including CKD [[13], [14], [15]]. Serum endotrophin levels independently correlate with mortality in CKD, suggesting that the accumulation of collagen VI and its cleavage product, endotrophin, promote mortality risk associated with CKD [13]. Another study demonstrated that urinary endotrophin levels predict renal fibrosis and disease progression in patients with CKD [14]. Recently, Karsdal et al. analyzed the association between serum endotrophin levels and various chronic diseases, and discovered that CKD mortality shows the strongest correlation with circulating endotrophin levels [15]. All the above evidence prompted us to propose endotrophin as a novel target for treatment of CKD. In addition to renal fibrosis, endotrophin is also suggested to mediate adipose tissue fibrosis in obesity [16], and chronic liver disease [17]. Moreover, blockade of endotrophin through neutralizing antibody potently suppresses tumor progression [11,18] and ameliorates adipose tissue fibrosis [16]. However, the therapeutic potential of endotrophin neutralizing antibodies in renal dysfunction and fibrosis has not yet been determined.

In our present study, our podocyte ablation model, the POD-ATTAC mouse, was utilized to assess the potential beneficial effects of endotrophin neutralizing antibodies. We demonstrate that neutralization of endotrophin significantly suppresses the pro-inflammatory and pro-fibrotic gene program, restores normal renal structure, decreases proteinuria, and eventually improves renal function. As part of our assessment of the beneficial effects of endotrophin antibodies in this podocyte injury model, we also utilize a lineage tracing system to confirm that podocytes can indeed regenerate *de novo*, upon podocytes injury and loss, and the blockade of endotrophin efficiently promotes podocyte regeneration in this POD-ATTAC model.

## 2 Material and methods

### 2.1 Animal models

All animal experiments were conducted using littermate-controlled male mice at an age of 8–10 weeks. Mice were maintained on a C57BL/6J background and housed in a barrier specific pathogen-free animal facility with a 12-hour dark–light cycle and free access to water and diet. Unless indicated otherwise, mice were fed standard chow diet (#5058, LabDiet, St. Louis, MO, USA). Doxycycline (dox)-containing pellet chow diet (600 mg/kg; S4107, Bio-Serv, Flemington, NJ, USA) was utilized in the lineage tracing studies. For the experimental cohorts on special diets, both control and transgenic mice were fed the same diet to control for the impact of the Dox.

*“Pod-Chaser” mouse model:* The transgenic construct (∼180 kilobases) expressing reverse tetracycline transcriptional activator (rtTA) under the control of the *Nphs2* promoter (*Nphs2*-rtTA) was generated by the bacterial artificial chromosome (BAC)-recombineering method (details see below). In total, ten founders were identified by PCR and one founder was chosen for further study based on inducing significant level of *Cre* mRNA only in the kidney but not in other tissues. After validation, the *Nphs2*-rtTA mouse was crossed with tetracycline responsive element-Cre transgenic mice (*TRE*-Cre; B6.Cg-Tg(tetO-cre)1Jaw/J, No. 006234, JAX) and Rosa26R-mT/mG (mT/mG, Gt(ROSA)26Sor^tm4(ACTB-tdTomato,-EGFP)Luo^/J, No. 007576, JAX) to generate the “Pod-Chaser” mice.

*BAC Recombineering to Derive Nphs2-rtTA transgene*: *Nphs2* homology arms corresponding to the first 50 bp upstream and the first 50 bp immediate downstream of the initiation ATG in Exon 2 were added to the forward and reverse primers, respectively, that amplified the rtTA-FRT-NEO-FRT sequence in the plasmid pL451-rtTA-FRT-Neo/Kan-FRT. The resulting PCR product was electroporated into SW105 cells carrying BAC RP23-387A22 that contains the full *Nphs2* locus. Homologous recombination of the resulting kanamycin/chloramphenicol resistant clones was confirmed by PCR. The Neo cassette was removed by l-arabinose induction of flp recombinase, resulting in bacteria carrying the final *Nphs2*-rtTA plasmid. Homologous recombination was further confirmed by sequencing. Then the whole Nphs2-rtTA BAC DNA was amplified and extracted through maxiprep for oocyte microinjection performed by the UT Southwestern Transgenic Core.

*POD-ATTAC mouse model*: The “POD-ATTAC” (podocyte-apoptosis through targeted activation of caspase-8) mouse model was generated and reported by our group previously. Dimerizer (0.4 μg/g body weight) was injected (i.p.) to induce podocyte apoptosis and loss.

### 2.2 Antibodies and reagents

Anti-podocin antibody (NBP2-75624) was obtained from Novus Biologicals (Littleton, CO, USA). Anti-GFP antibody (ab13970) was from Abcam (Cambridge, MA, USA). Anti-GAPDH (#5174S) antibody was from Cell Signaling Technology, Inc. (Beverly, MA, USA).

*Preparation of endotrophin neutralizing antibody ENT4-Mu*: The parental ENT4 was isolated from a single memory B cell from a rabbit immunized with the human endotrophin protein recombinantly expressed in HEK293 cells [18]. To avoid immunogenicity against the rabbit antibody during long term and multiple dosing treatment in mice, we mouserized the parental rabbit ENT4 antibody. Mouserization was accomplished by using a combined KABAT/IMGT CDR-grafting method. The VH and VL DNA sequences of the parental antibody were blasted against the mouse germline gene sequence database with IgBLAST or IMGT/V-QUEST. The most similar mouse germline VH and VL sequences were selected as templates. The CDRs defined by KABAT/IMGT were grafted onto the framework regions of corresponding templates. The CDR-grafted VH and VL were cloned into mouse IgG1 and light chain backbone to express the full-length antibody. Antibody expression and purification were based on protocols described previously. Briefly, the antibody was produced by transient transfection of Expi293 with PEI-max and feed-batch cultivation for 7 days. The antibody was purified on Protein-A resin column. All solutions used in purification were made with endotoxin-free water.

Unless specifically indicated, all other reagents were obtained from Millipore Sigma Corporation (St. Louis, MO, USA).

### 2.3 Determination of relative podocyte number in “Pod-Chaser” mice

For lineage tracing in the “Pod-Chaser” mice, relative podocyte numbers were determined through the following labeling strategy [19]:

Total podocyte number per glomerulus: GFP^+^/podocin^+^ cells plus GFP^−^/podocin^+^ cells per glomerulus at Day 0
Recovered podocytes per glomerulus: (Total podocyte number on Day 60) − (Average total podocyte number on Day 2) per glomerulus
*De novo* regenerated podocytes: GFP^−^/podocin^+^ cells per glomerulus
Percentage of newly generated podocytes:
(GFP^−^/podocin^+^ cell number on Day 60)∗100/[(Total podocyte number on Day 60) − (Average total podocyte number on Day 2)]

For calculations and statistics, at least 30 glomeruli in each kidney section were selected randomly and counted, and each timepoint has three mice.

### 2.4 Histological analysis

Mouse kidneys in each group were paraffin-embedded, sectioned at 4 μm, and stained with hematoxylin and eosin (H&E) or Trichrome. The staining was performed by the UT Southwestern Pathology Core. Images (100× or 200× magnification) were taken on a Keyence BZ-X microscopy (Keyence, Itasca, IL, USA). In all cases, representative histological images are shown.

### 2.5 Immunofluorescence staining

Paraffin-embedded slides were prepared by the UT Southwestern Histology Core for immunostaining. Briefly, deparaffinized kidney sections were stained with anti-podocin (1:200) and anti-GFP (1:700) primary antibodies and incubated overnight at 4 °C. Then the corresponding fluorescent-labelled secondary antibodies (Life Technologies) were added, and finally the sections were counterstained with DAPI before cover slips were added. The fluorescence images were acquired with a Keyence BZ-X Microscope.

Collagen staining was performed using a Collagen Hybridizing Peptide staining kit (#5276, Advanced BioMatrix, Carlsbad, CA, USA), following the instructions. Briefly, deparaffinized kidney sections were blocked with 5% BSA and then incubated with diluted Collagen Hybridizing Peptide (20 μM) at 4 °C for 2 h. After washing, the sections were counterstained with DAPI before cover slips were added. The sulfo-Cyanine3 (CY3) fluorescence images were acquired with a Keyence BZ-X Microscope.

### 2.6 RNA extraction and real-time quantitative PCR (qPCR)

Mice were sacrificed and kidneys and other tissues were collected, snap-frozen in liquid nitrogen, and stored at −80 °C until further processing. For total RNA extraction, the tissues were homogenized in TRIzol (Invitrogen, Carlsbad, CA, USA) using a TissueLyser (Qiagen, Valencia, CA, USA) and RNA was extracted using the RNeasy RNA Extraction Kit (#74106, Qiagen, Valencia, CA, USA). The quality and concentration of RNA were determined using a BioTek Take3 Microplates at the Epoch Microplate Spectrophotometer (BioTek, Agilent, Santa Clara, CA, USA). A total of 1 μg RNA was reverse transcribed using the iScript cDNA Synthesis Kit (#170-8891, Bio-Rad Laboratories, Inc., Hercules, CA, USA). cDNAs were diluted to 5 ng/μl and stored at −20 °C until further use. SYBR Green PCR Master Mix (A25742, Life Technologies, Carlsbad, CA, USA) was utilized to perform qPCR reactions on a QuantStudio 6 Flex Real-Time PCR System (Applied Biosystems, Foster City, CA, USA). All primer sequences used in this study were designed through Primer-BLAST on NCBI, validated in previous studies. Mouse *Gapdh* was used as internal controls for relative quantification of gene expressions.

### 2.7 Serum chemistry

Mice were sacrificed and whole blood was collected and centrifuged for 15 min at room temperature at 6,000 rpm to obtain the serum. Samples were analyzed for blood urea nitrogen (BUN), sodium, and potassium using a Vitros 250 Chemistry System (Ortho Clinical Diagnostics, Raritan, NJ, USA) at the UT Southwestern Metabolic Phenotyping Core.

### 2.8 Western blot analysis

Frozen tissue samples were homogenized, and cells were lysed in RIPA buffer (50 mM Tris–HCl, 250 mM NaCl, 5 mM EDTA, and 1% Triton-X100; pH 8.0) containing PMSF (phenylmethylsulfonyl ﬂuoride) and 1× Protease Inhibitor Cocktail (Calbiochem, San Diego, CA, USA). Samples were centrifuged for 15 min at 12,000 *g* and 4°C, supernatants were collected, and protein concentrations were measured using the BCA kit. For immunoblotting, protein samples (30 μg) were separated on a 4–12% SDS-PAGE gel. Next, proteins were transferred to a nitrocellulose membrane and non-specific binding sites were blocked by incubation in TBS-T (20 mM Tris–HCl, 137 mM NaCl, and 0.05% Tween-20; pH 7.6) containing 5% BSA. The membranes were then incubated overnight at 4 °C with specific primary antibodies: anti-podocin (1:1000), and anti-GAPDH (1:1000). The membranes were washed, appropriate fluorescent-conjugated secondary antibodies (IRDye, LI-COR, Lincoln, NE, USA) were added, and the membranes were incubated for 1 h at room temperature. The membranes were washed again and scanned on a LI-COR Odyssey Imager (LI-COR, Lincoln, NE, USA). Band intensity was analyzed using LI-COR Odyssey Imager software.

For determination of proteinuria by Western Blot, diluted urine samples (1:100 in TBS-T) were separated on SDS-PAGE gels and proteins were transferred to a nitrocellulose membrane. The membrane was then stained with Ponceau S for 5 min and images were taken for further analysis.

### 2.9 Statistics

All data are presented as means ± SEM of biological replicates. GraphPad Prism 8.4.3 (GraphPad Software, Inc., La Jolla, CA, USA) was used for statistical analyses. One-way analyses of variance (ANOVAs) followed by Tukey post-tests were used for comparisons between more than two independent groups. *P* < 0.05 was considered statistically significant.

### 2.10 Study approval

All animal experiments and procedures were approved by the Institutional Animal Care and Use Committee (IACUC) of The UT Southwestern Medical Center at Dallas, Texas (APN# 2015-101207).

## 3 Results

### 3.1 Blockade of endotrophin improves renal function, fibrosis, and proteinuria

**Figure 1:**
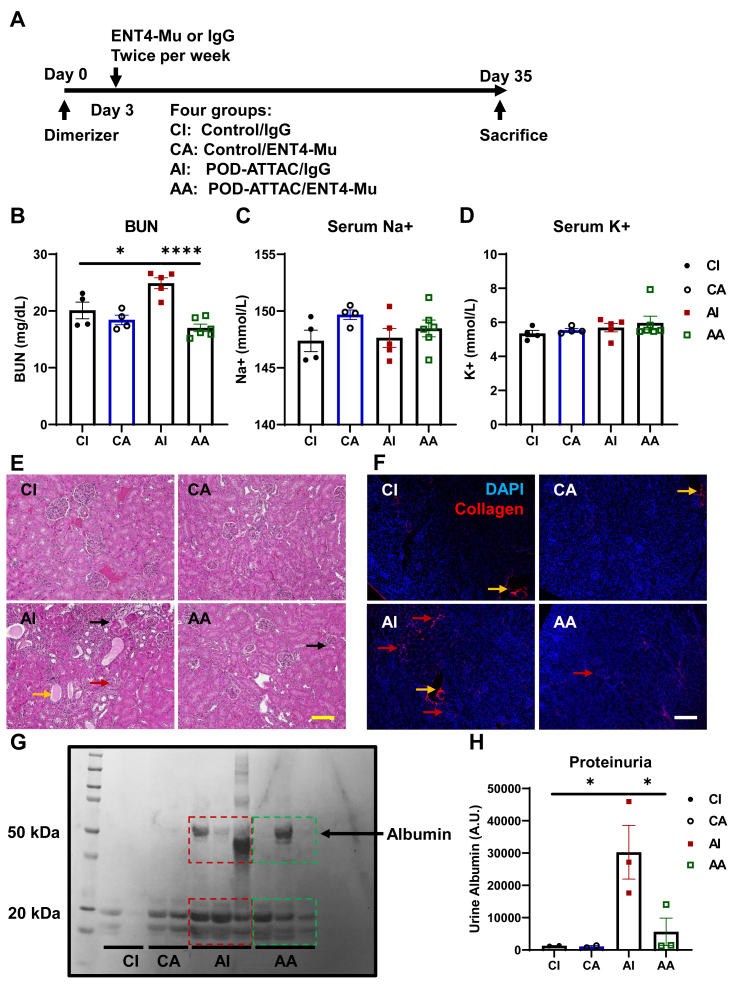
**Blockade of endotrophin by ENT4-Mu improves renal function, fibrosis, and proteinuria.** (**A**) Schematic illustration of the treatment procedures. 0.4 μg/g (body weight) dosage of dimerizer was injected at Day 0, and then either control IgG or ENT4-Mu antibody (100 μg/mouse) was given to either control or POD-ATTAC mice starting from Day 3. Four groups are shown here: CI, n = 4; CA: n = 4; AI, n = 5; AA, n = 6. (**B**–**D**) Blood urea nitrogen (BUN) (**B**), serum sodium (Na^+^) (**C**), and serum potassium (K^+^) (**D**) levels in four groups. (**E**) Kidney H&E staining images, bar = 161 μm. Arrows indicate inflammatory cell infiltration (black arrow), tubule casts (yellow arrow), and glomerular cell death (red arrow). (**F**) Kidney immunofluorescence staining of DAPI and denatured collagen (DAPI: blue, collagen: red), bar = 161 μm. Arrows indicate collagen deposition (red arrow), and unspecific blood vessel staining (yellow arrow). (**G**–**H**) Representative Ponceau S staining image (**G**) of blots from urine samples from four groups and statistics for urine albumin (**H**). Data are presented as mean ± SEM of biologically independent samples. ∗*P* < 0.05, ∗∗∗∗*P* < 0.0001. One-way ANOVA followed by a Tukey post-test (**B**–**D**, **H**).

To directly evaluate the therapeutic potential of endotrophin neutralizing monoclonal antibodies on renal function and fibrotic parameters during chronic kidney diseases, control or POD-ATTAC mice subject to dimerizer induction (Day 0) were administered twice a week with intraperitoneal (i.p.) injections of either an isotype-adjusted IgG control or the monoclonal anti-mouse endotrophin antibody, clone ENT4-Mu. The screening and full characterization of ENT4 antibody in its endotrophin-neutralizing ability, *in vivo* half-life, and its anti-breast cancer growth and metastasis activity has been reported by our group [18]. IgG control or ENT4-Mu antibody treatments were firstly administered 3 days post-dimerizer and were continued for 5 weeks after the initial dimerizer induction and podocyte insult (Figure 1A). The production of the ENT4 antibody and the characterization of its neutralization have been described in our previous publication [18]. Upon sacrificing mice on Day 35, we evaluated the renal function through measuring blood urea nitrogen (BUN) and serum electrolyte levels. As shown in Figure 1B, POD-ATTAC mice receiving IgG injection (AI group) demonstrated higher BUN levels, and by contrast, treatment of ENT-Mu antibody significantly decreased BUN levels, indicating an improved renal function after podocyte injury and loss. Serum sodium and potassium levels maintained identical among four different groups (Figure 1C–D).

To demonstrate whether the endotrophin neutralizing antibody improves the renal architecture in POD-ATTAC mice, kidney sections were subject to further histological analyses. By H&E staining, we demonstrate that podocyte injury results in significant inflammatory cell infiltration, cast formation in the tubules, and glomerular cell death (AI group). However, treatment of ENT4-Mu antibody ameliorated majority of the pathological changes above (AA group) (Figure 1E). We performed a collagen stain using a sensitive peptide probe binding to denatured collagen strands to assess renal fibrotic changes. As shown in Figure 1F, a significant deposition of collagens surrounding the tubules and glomeruli was demonstrated in POD-ATTAC mice receiving IgG injections, but POD-ATTAC mice receiving ENT4-Mu injections demonstrated much lower levels of collagen hybridizing signal and accumulation in the kidney.

Histologically, neutralization of endotrophin maintains kidney structure and decreases renal collagen deposition. We evaluated whether endotrophin neutralizing antibodies show benefits with regards to maintaining renal filter barrier function. Proteinuria is a common consequence of podocyte injury and loss, and as expected, we indeed observed a significant elevation of albumin levels in the urine of POD-ATTAC mice with IgG treatment (Figure 1G). Consistent with the morphological improvements, ENT4-Mu antibody treatment significantly reduced urinary protein levels (Figure 1G–H), suggesting that blocking endotrophin action also improves renal function, fibrosis, and proteinuria.

### 3.2 Blockade of endotrophin suppresses inflammatory and fibrotic responses to podocyte injury

Endotrophin plays a significant role in augmenting tissue inflammation and fibrosis in various tissues. Upon podocyte injury, inflammatory cell infiltration, especially macrophage accumulation, further exacerbates the tissue damage, and leads to the ultimate consequence of podocyte injury, i.e., fibrosis [20,21]. Therefore, we assessed a series of transcriptional changes to evaluate the impact of endotrophin neutralization on inflammatory and fibrotic gene expression.

**Figure 2:**
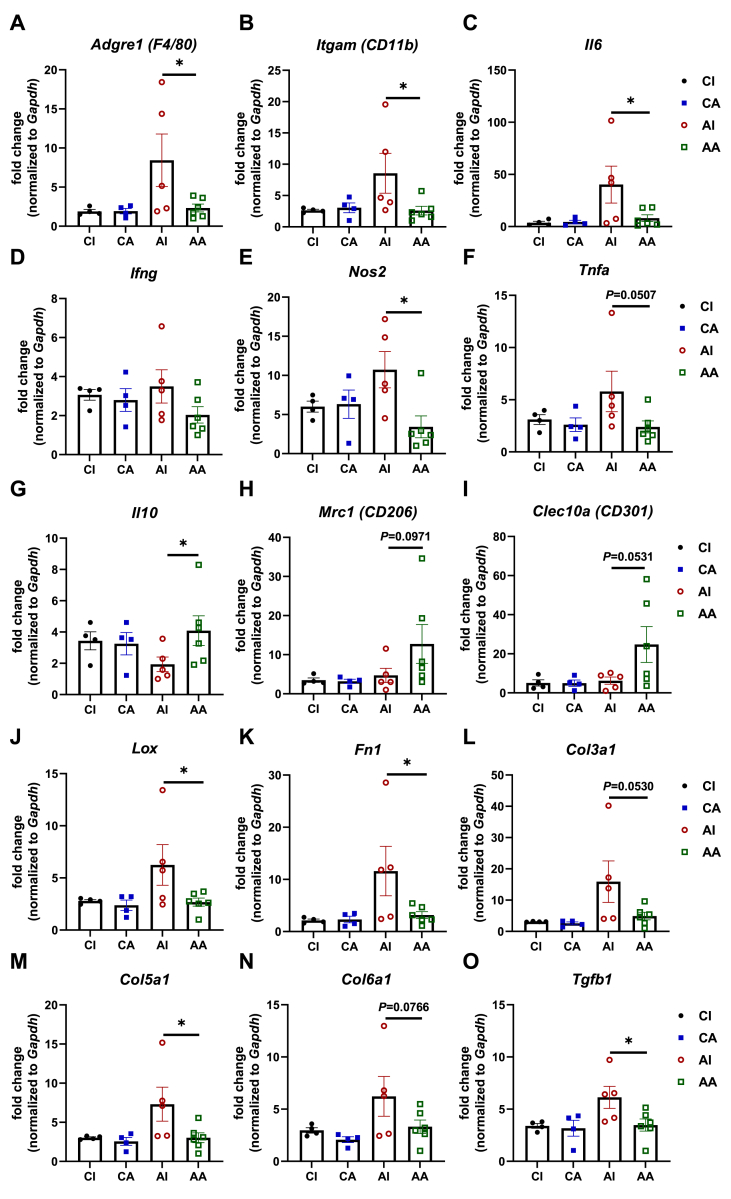
**Blockade of endotrophin by ENT4-Mu reduces renal inflammation through promoting an M2-like macrophage polarization and suppresses renal fibrotic gene programs.** (**A**–**I**) Selective inflammation related gene expression in kidney macrophages: general macrophage markers, *Adgre1*, *Itgam*, and *Il6;* M1 markers, *Ifng*, *Nos2*, and *Tnfa*; M2 markers, *Il10*, *Mrc1*, and *Clec10a*. (**J**–**O**) Selective fibrosis related gene expression in kidneys from four groups: *Lox*, *Fn1*, *Col3a1*, *Col5a1*, *Col6a1*, and *Tgfb1*. Data are presented as mean ± SEM of biologically independent samples. ∗*P* < 0.05. One-way ANOVA followed by a Tukey post-test (**A**–**O**).

We assessed several general macrophage markers, *Adgre1*, *Itgam*, and *Il6*. We found that while podocyte injury results in the expected elevated macrophage marker expression, the ENT4-Mu antibody treatment reduces all three general marker genes for macrophages to baseline levels (Figure 2A–C). Recent observations in the literature point to the presence of differentially polarized macrophages that play distinct roles in chronic kidney disease progression [21,22]. Particularly, the M1-like macrophage population exerts a pro-inflammatory and injury promoting role; by contrast, the M2-like macrophage population exerts an anti-inflammatory and injury resolving role. Thus, we assessed the relative degree of macrophage polarization and measured markers for M1-like and M2-like polarization in the kidneys. Most of the assessments failed to reach statistical significance, leading us to conclude that there is limited impact on macrophage distribution. However, there is significant scatter with respect to these markers, but trends are clearly apparent. As for the M1-like pro-inflammatory markers, we found that neutralization of endotrophin tends to suppress *Ifng* and *Tnfa* levels, and significantly reduces *Nos2* (Figure 2D–F). In contrast, the ENT4-Mu antibody significantly augments the M2 anti-inflammatory marker *Il10*, with trends towards increase *Mrc1*, and *Clec10a* (Figure 2G–I). Collectively, we demonstrated that blocking endotrophin action suppresses overall macrophage infiltration upon podocyte injury and promotes a shift from M1 to M2 macrophages, resulting in a less pro-inflammatory microenvironment.

The endpoint of podocyte injury and renal inflammation is sclerosis. Therefore, we assessed gene expression of critical players that contribute to the fibrotic process during chronic kidney disease. As shown in Figure 2J–O, we observed that POD-ATTAC mice treated with IgG control antibodies display a significantly higher level of pro-fibrotic factors, such as *Lox* and extracellular matrix markers, such as *Fn1*, *Col3a1*, *Col5a1*, *Col6a1*, and the prominent transcriptional regulator for fibrosis, *Tgfb1*. Administrating ENT4-Mu antibodies to POD-ATTAC mice effectively suppressed the whole fibrotic gene program and lowered it down to baseline levels. Combined with the histological evidence shown in Figure 1F, and the transcriptional assessment of pro-fibrotic genes leads us to conclude that blocking endotrophin action significantly ameliorates renal fibrosis induced by podocyte injury.

In addition to the results obtained above by utilizing the endotrophin neutralizing antibody clone ENT4-Mu, we also injected another clone 10B6 (first injection on Day 3 post dimerizer and continuation for 5 weeks) to POD-ATTAC mice (Fig. S1A). The monoclonal 10B6 shows similar neutralizing activity for endotrophin and exerts potent anti-fibrotic actions in adipose tissues in our previous report [16]. Consistent with the findings that we have observed in ENT4-Mu treated mice, POD-ATTAC mice receiving 10B6 antibodies significantly improved renal structure and integrity assessed by H&E staining (Fig. S1B), decreased collagen deposition assessed by Trichrome staining (Fig. S1C), and downregulated fibrotic gene transcription, such as *Lox*, *Col3a1*, and *Fn1* (Fig. S2).

### 3.3 Blockade of endotrophin potentially promotes podocyte regeneration

The current POD-ATTAC model that we utilized in assessing the therapeutic potential of endotrophin antibody mimics the central event in chronic kidney disease, podocyte injury. However, the major consequence of podocyte injury results in glomerulosclerosis, one form of renal fibrosis. In order to further evaluate the anti-fibrotic property of endotrophin antibody and expand its potential usage, we went on to determine the efficacy of ENT4-Mu antibody in the *Unilateral Ureteral Obstruction (UUO) model* (Fig. S3A). The UUO-obstructed kidney is characterized by tubular dilation, tubule epithelial cell death, and fibroblast activation, finally leading to progressive renal tubulointerstitial fibrosis [23]. Surprisingly, unlike the significant improvement in renal function and fibrosis in the POD-ATTAC model, neutralization of endotrophin by ENT4-Mu antibody was not able to restore the elevation of blood urea nitrogen levels (Fig. S3B), and but showed a significant effect on correcting serum potassium levels (Fig. S3C). Although ENT4-Mu treatment could still promote an M1 to M2 macrophage polarization transition, the overall macrophage infiltration maintained at a high level even after endotrophin neutralization, suggested by higher *Adgre1* and *Itgam* expressions (Fig. S4). More importantly, UUO challenged mice with or without ENT4-Mu antibodies demonstrated identical expression levels for fibrotic markers (Fig. S5), suggesting that blockade of endotrophin has no impact on tubulointerstitial fibrosis induced by the UUO model.

It is interesting that the improvement by endotrophin neutralization on renal fibrosis is much more effective with podocyte injury-induced glomerular fibrosis. This phenomenon prompted us to hypothesize that blockade of endotrophin might have a direct impact on podocyte regeneration after injury.

However, before we could evaluate whether the endotrophin antibody affects podocyte regeneration, we needed to establish a reliable podocyte-specific tool mouse that allows us to monitor the process with high resolution. Previous lineage tracing studies show discrepancies in determining the sources of regenerated podocytes: while several studies argue that regenerated podocytes could come from redifferentiation of dedifferentiated podocytes [[24], [25], [26]], others argue that the majority of regenerated podocytes are *de novo* differentiated podocytes derived from progenitor cell populations [19,[27], [28], [29], [30]]. Furthermore, previous lineage tracing experiments mostly relied on the tamoxifen-inducible system, and our group has previously shown that residual tamoxifen enables the Cre recombinase to translocate into the nucleus even after a long washout period (e.g., two months in adipocytes) [31]. This limitation may weaken the temporal resolution of the system with tamoxifen, and may give rise to different lineage tracing outcomes depending on lengths of the washout periods. To overcome these difficulties, we decided to generate a full podocin gene (*Nphs2*) promoter driven reverse tetracycline-controlled transactivator (rtTA) (*Nphs2-rtTA*) mouse line that allows us to take advantage of a doxycycline (Dox)-inducible system. By crossing the Nphs2-rtTA together with TRE-Cre and membrane TdTomato/membrane green fluorescent protein (GFP) (mT/mG) mice, we generated a reporter mouse (“Pod-Chaser”) that enables us to label pre-existing podocytes as green (GFP^+^) and podocytes *de novo* differentiated post injury as red (Tomato^+^) in a genetic “pulse-chase” experiment by adding and removing Dox. In contrast to tamoxifen, doxycycline washes out of the system within 8–12 h. As shown in Fig. S6, with a brief period of labeling (one week of Dox) and washout (1 week of removing Dox), we observed a clear GFP^+^ signal in the glomerulus and an almost 100% overlap with the podocyte specific marker, podocin (Fig. S6B). We also demonstrated by co-staining that the GFP signaling overlays with another podocyte specific molecule, nephrin (*data not shown*). Above results implicate that the “Pod-Chaser” mouse efficiently labels pre-existing podocytes and is suitable to determine the source of regenerated podocytes in a podocyte ablation model.

**Figure 3:**
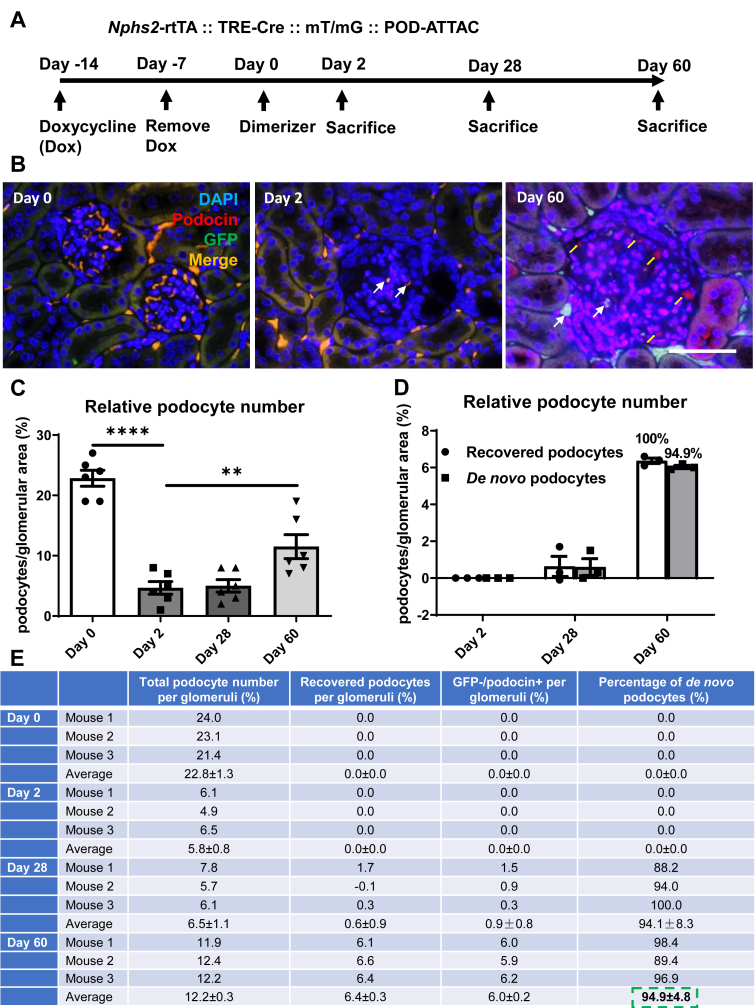
**Lineage tracing evidence supports de novo regeneration of podocytes in POD-ATTAC model.** (**A**) Schematic illustration of the lineage tracing procedures. Before 0.4 μg/g (body weight) dosage of dimerizer injection at Day 0, doxycycline (Dox) was given to mice at Day −14 and removed at Day −7. After Dox washing out for 7 days, dimerizer was injected and mouse kidneys were harvested at Day 0, 2, 28, and 60. White arrow indicates preexisting podocytes (GFP^+^/podocin^+^ cells), and yellow arrow indicates *de novo* regenerated podocytes (GFP^−^/podocin^+^ cells). (**B**) Kidney immunofluorescence staining of DAPI, podocin, and GFP (DAPI: blue; podocin: red; GFP: green; merge: orange), bar = 161 μm. (**C**–**E**) Calculation and statistics of relative podocyte number per glomeruli and the percentage of *de novo* regenerated podocytes (GFP^−^/podocin^+^ cells), n = 3 for each timepoint. For all the statistics: data are presented as mean ± SEM of biologically independent samples. ∗∗*P* < 0.01, ∗∗∗∗*P* < 0.0001. One-way ANOVA followed by a Tukey post-test (**C**).

Therefore, we further crossed the “Pod-Chaser” mice with the POD-ATTAC mice to monitor the ablation and regeneration of podocytes in this lineage tracing model (Figure 3A). Two days post dimerizer injection, we noticed a severe loss of podocytes in POD-ATTAC mice. On Day 60, we discovered enhanced podocin^+^ cells located in the glomerulus (Figure 3B), suggesting the regeneration of podocytes in the POD-ATTAC ablation model. Statistically, at baseline on Day 0, the average podocyte number per glomerulus is 22.8 ± 1.3%, and on Day 60 after dimerizer insult, the podocyte number rises to 12.2 ± 0.3%, showing a significantly higher value than Day 2 (5.8 ± 0.8%, *P* < 0.01) (Figure 3C). Furthermore, Figure 3B demonstrated that on Day 60, regenerated podocytes were mostly podocin^+^ cells without overlay with a GFP^+^ staining, implicating regenerated podocytes are unlikely derived pre-existing podocytes, but rather they are *de novo* differentiated podocytes. Utilizing a more detailed quantitative method, when we define *de novo* podocytes as GFP^−^/podocin^+^ cells, we could confirm that 94.9 ± 4.8% of regenerated podocytes in the POD-ATTAC model derived from *de novo* podocyte differentiation (Figure 3D–E).

**Figure 4:**
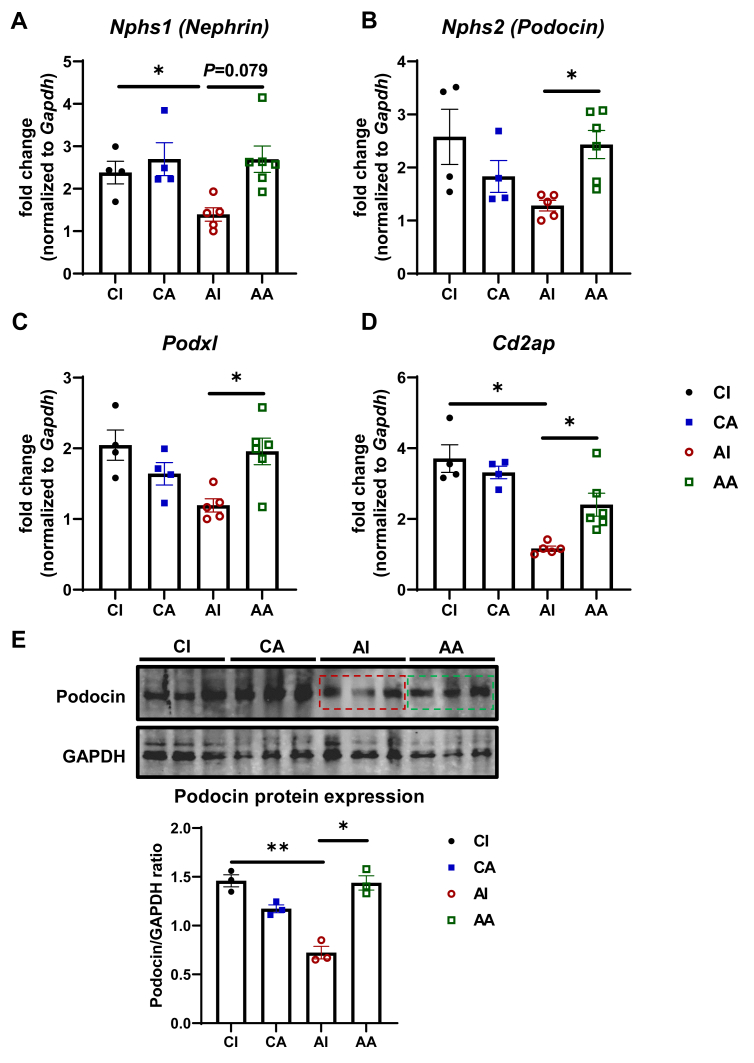
**Blockade of endotrophin by ENT4-Mu promotes podocyte marker expressions.** (**A**–**D**) Selective podocyte marker gene expressions in kidneys from four groups: *Nphs1*, *Nphs2*, *Podxl*, and *Cd2ap*. (**E**) Representative Western Blotting image of podocin and its statistics in four groups. Data are presented as mean ± SEM of biologically independent samples. ∗*P* < 0.05. One-way ANOVA followed by a Tukey post-test (**A**–**D**).

We confirm that podocytes could be *de novo* regenerated after podocyte injury and loss using the above lineage tracing model, implicating modulating podocyte regeneration is a viable strategy to ameliorate podocyte injuries. Thus, we moved forward to evaluate the impact of anti-endotrophin antibodies on podocyte regeneration and markers. At a transcriptional level, POD-ATTAC mice administrated with IgG showed a significant downregulation of podocyte markers [32], including *Nphs1* (encoding Nephrin), *Nphs2* (encoding Podocin), *Podxl*, and *Cd2ap*, consistent with the severe podocyte injury and ablation phenotypes (Figure 4A–D). By contrast, treatment of ENT4-Mu antibody significantly upregulated these podocyte specific gene expressions (Figure 4A–D), suggesting that neutralizing endotrophin antibodies enhance podocyte regeneration. The enhanced regeneration of podocytes by endotrophin neutralization was also validated at the protein level, and we observed that ENT4-Mu treatment significantly restores podocin protein levels compared to the IgG treated POD-ATTAC group (Figure 4E).

## 4 Discussion

Our present study offers the first evidence invoking endotrophin as a major contributor to renal fibrosis, based on the observations that neutralizing endotrophin through targeted antibody treatment suppresses renal fibrosis in a podocyte injury-induced CKD mouse model. A lot of efforts over the past years have led to delineating novel mechanisms associated with renal fibrosis and the identification of possible targets. However, so far, very few of these discoveries have made it to the clinic [33]. Here, we present data highlighting a very promising therapeutical approach allowing to ameliorate and even reverse renal fibrosis. We show that a 5-week treatment of endotrophin antibody post podocyte injury dramatically suppresses inflammatory and fibrotic gene expression, restores kidney architecture, inhibits collagen deposition, decreases proteinuria, and improves renal function. These data direct us to consider our pre-clinical mouse models for use to further evaluate the therapeutic impact of neutralizing antibodies, such as determining appropriate dosage, additional pharmacological parameters, and the pharmacokinetics of endotrophin neutralizing antibodies prior to moving forward to studies in humans. In addition, the endotrophin neutralization is not only promising in renal fibrosis approaches, but also shows significant benefits in other fibrotic diseases. Neutralizing monoclonal antibodies against mouse or human endotrophin effectively curb tumor growth and enhance chemosensitivity in either mouse or human breast cancer models, respectively [11,18]. Blocking endotrophin also reverses adipose tissue inflammation and fibrosis, ameliorates metabolic defects under a high-fat diet exposure, and effectively improves obesity and associated metabolic diseases [16]. Combined, endotrophin neutralization is emerging as a viable anti-fibrotic strategy that can be applied in renal fibrosis, as well as fibrosis in other organs and in tumors.

The other point we are making in our current study is that we provide a quantitative conclusion in podocyte regeneration post podocyte ablation using our newly developed lineage tracing model, the “Pod-Chaser” mouse. Podocytes are highly differentiated and specialized cells, and are commonly considered terminally differentiated and unable to self-renew. Here, we utilize our lineage tracing model to confirm that podocytes can be regenerated upon applying an intermediate dose of dimerizer in the POD-ATTAC podocyte injury model. By day 60 post insult, we observe approximately 24.9% of podocytes regenerating compared to the severe podocyte ablation on Day 2. In addition, by detailed quantitative assessment, we demonstrate that 94.9% of the regenerated podocytes are *de novo* differentiated podocytes derived from stem/progenitor cells. Importantly, they do not arise from cell division of pre-existing podocytes (proliferation or dedifferentiation and redifferentiation). Our conclusion is consistent with lineage tracing studies using tamoxifen-inducible tools or doxycycline-inducible tools by other groups, and confirms *de novo* podocyte genesis is the major form of podocyte regeneration [19,20,30]. Our lineage tracing system offers superior quantitative power because our newly generated *Nphs2*-rtTA mouse is produced through the bacterial artificial chromosome (BAC)-based transgenic strategy and the full promoter for the *Nphs2* gene is included. Therefore, we are able to label pre-existing podocytes close to 100%. In an equivalent manner, our group has also developed similar lineage tracing systems in other tissues or cell types, such as the “Adipo-Chasers” for mature adipocytes [34,35], “Alpha-Chasers” and “Beta-Chasers” for pancreatic β-cells [36], and “Mural-Chasers” for adipose progenitor PDGFβR^+^ cells [37]. Furthermore, previous lineage tracing reports in various podocyte ablation models have led to discrepant findings with regards to the identification of the specific progenitors that are responsible for giving rise to *de novo* podocyte genesis in adults. There is evidence for and against glomerular parietal epithelial cells (PEC) and renin^+^ cells as podocyte progenitors [19,29,38,39]. Future efforts in the lab are directed to utilize our “Pod-Chaser” and POD-ATTAC mouse models to discover *bona fide* podocyte progenitors that account for *de novo* podocyte formation and renal function recovery.

Several unresolved questions will need to be addressed in the future. First, how does endotrophin mediate its actions in the various tissues. As a major component and inducer of the extracellular matrix (ECM), endotrophin is elevated under many stress conditions, such as obesity and podocyte injury, and its role may be to assist in the remodeling of the ECM. The other contributing factor may be that endotrophin could serve as a chemoattractant for macrophages to induce a pro-inflammatory microenvironment and thereby endotrophin further promotes fibrosis. This can also explain why we consistently observe macrophage polarization changes upon neutralizing endotrophin in the various CKD models used, such as the POD-ATTAC mouse and UUO. In addition, endotrophin promotes the epithelial–mesenchymal transition (EMT) process, and thus mediates fibroblast activation and fibrotic pathogenesis. Future efforts are required to identify the exact downstream signaling pathways by which endotrophin mediates tissue fibrosis, and to determine whether tissue-specific mechanisms are at work. The reasons why endotrophin neutralization could not reduce fibrosis in the UUO model is not clear. However, UUO induces tubulointerstitial fibrosis and is a very aggressive model of kidney disease. Thus, the difference of disease progression between the UUO model and the POD-ATTAC model might account for the different effects of the antibody treatment. This indicates that endotrophin neutralizing antibodies may be more effective in human CKD at earlier stages and in a glomerular injury context. In addition, we do not understand how neutralization of endotrophin leads to enhanced podocyte regeneration after podocyte injury and loss. We conclude that the major benefit of endotrophin neutralization may be in the context of podocyte ablation-induced glomerulosclerosis, and is associated with its ability to enhance *de novo* podocyte-regeneration. This is consistent with a model that suggests that endotrophin actively suppresses recruitment of precursor cells, and the neutralization of endotrophin facilitates the recruitment of such precursors to reconstitute the pool of podocytes. However, at present, we do not know which progenitor cells endotrophin acts on. Future studies will need to be performed in our lineage tracing system (the “Pod-Chaser”) to assess the effects of endotrophin antibodies on podocyte numbers and regeneration. Furthermore, it is also necessary to better understand the relationship between renal fibrosis and enhanced podocyte regeneration: does the neutralization of endotrophin lead to reduced fibrosis which in turn creates a more supportive environment for podocyte regeneration?

We are also aware that limitations exist in the present study. Only a single dosage of endotrophin antibodies has been applied, and future studies are required to optimize the dosage in the context of renal fibrosis models. In addition, we have limited information regarding the sexually dimorphic responses for utilizing endotrophin antibodies to treat renal fibro-inflammatory chronic conditions, since we only used male mice in this study. However, our data from this study strongly support the therapeutic efficacy of this approach. While we did not observe any toxicity with regards to these antibodies, and all mice gained comparable amounts of weight during the treatment period, we will need to address any off-target effects more systematically prior to exploring this new treatment approach in a clinical setting.

## 5 Conclusion

While details still need to be worked out, we highlight here the major impact that endotrophin exerts on renal fibrosis and impaired renal function in the context of a podocyte injury model. Our study provides strong pre-clinical evidence supporting neutralizing endotrophin as a promising therapeutic avenue for reversing renal fibrosis in CKD, and suggests the possibility of expanding the use of endotrophin neutralizing antibodies in other fibro-inflammatory chronic diseases.

## Author contributions

Designing research studies: Y.A.A. and P.E.S.; conducting experiments: Y.A.A., S.C., D.B., J.M.R., and C.M.K.; acquiring data: Y.A.A. and S.C.; analyzing data: Y.A.A. and S.C.; providing reagents and neutralizing antibodies: W.X., N.Z., and Z.A.; writing and revising the manuscript: Y.A.A., J.P.B., C.M.K., Z.A., and P.E.S.

## Data Availability

Data will be made available on request.
