# Supplementary material for: Endotrophin neutralization through targeted antibody treatment protects from renal fibrosis in a podocyte ablation model
Source: Mol Metab. 2023 Jan 22;69:101680. doi: 10.1016/j.molmet.2023.101680 (PMC9918787; doi:10.1016/j.molmet.2023.101680)
Supplement: Multimedia component 1 [file mmc1.pdf]

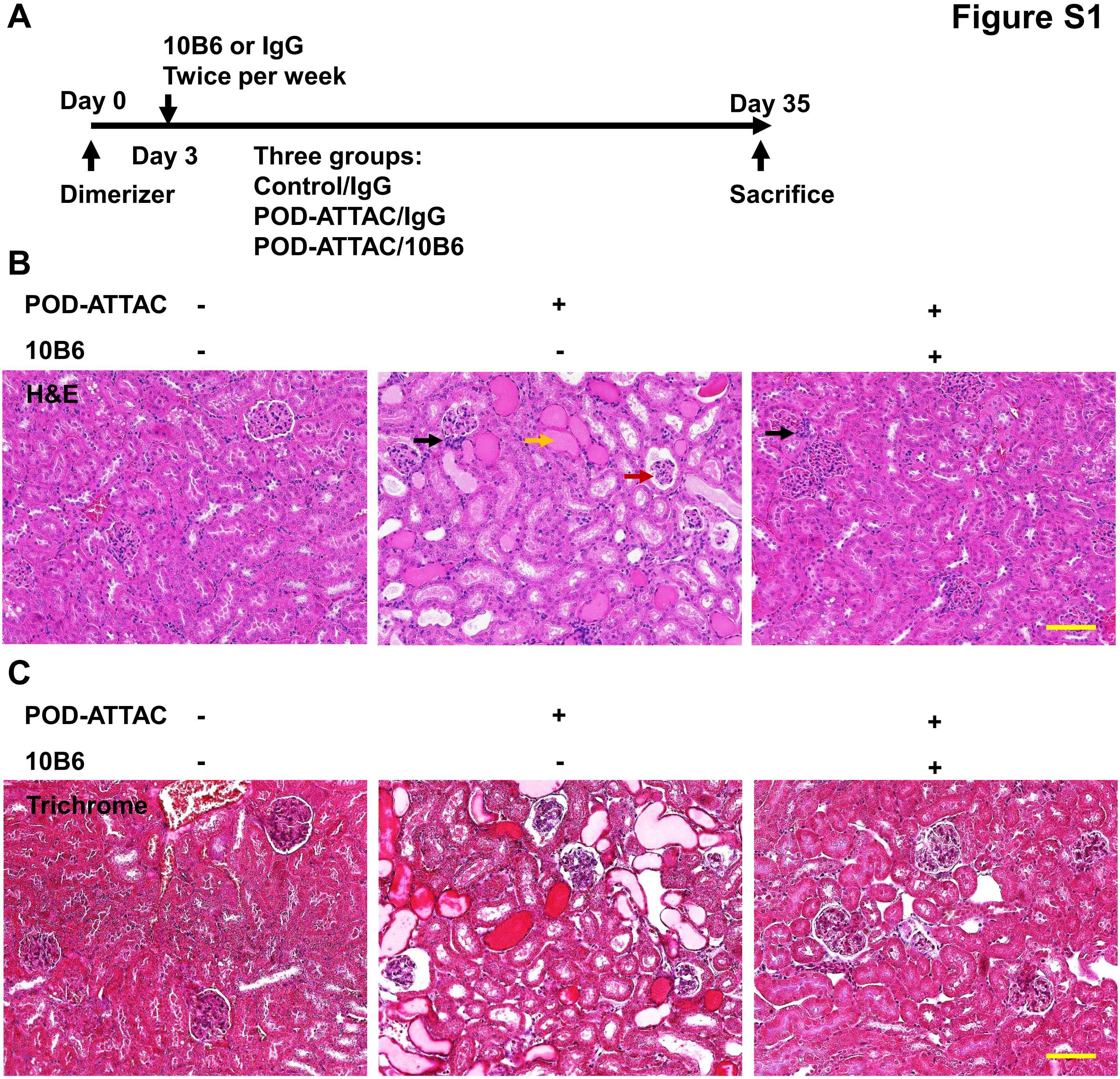

Fig. S1. Neutralization of endotrophin by 10B6 improves renal histology and fibrosis. (A) Schematic illustration of the treatment procedures. 0.4µg/g (body weight) dosage of dimerizer was injected at Day 0, and then either control IgG or 10B6 antibody (100µg/mouse) was given to either control or POD-ATTAC mice starting from Day 3. Three groups are shown here: Control/IgG, n=4; POD-ATTAC/IgG: n=3; POD-ATTAC/10B6, n=5. (B-C) Kidney H&E staining images (B) and Trichrome staining images (collagen: blue, C), bar = 161 µm. Arrows indicate inflammatory cell infiltration (black arrow), tubule casts (yellow arrow), and glomerular cell death (red arrow).

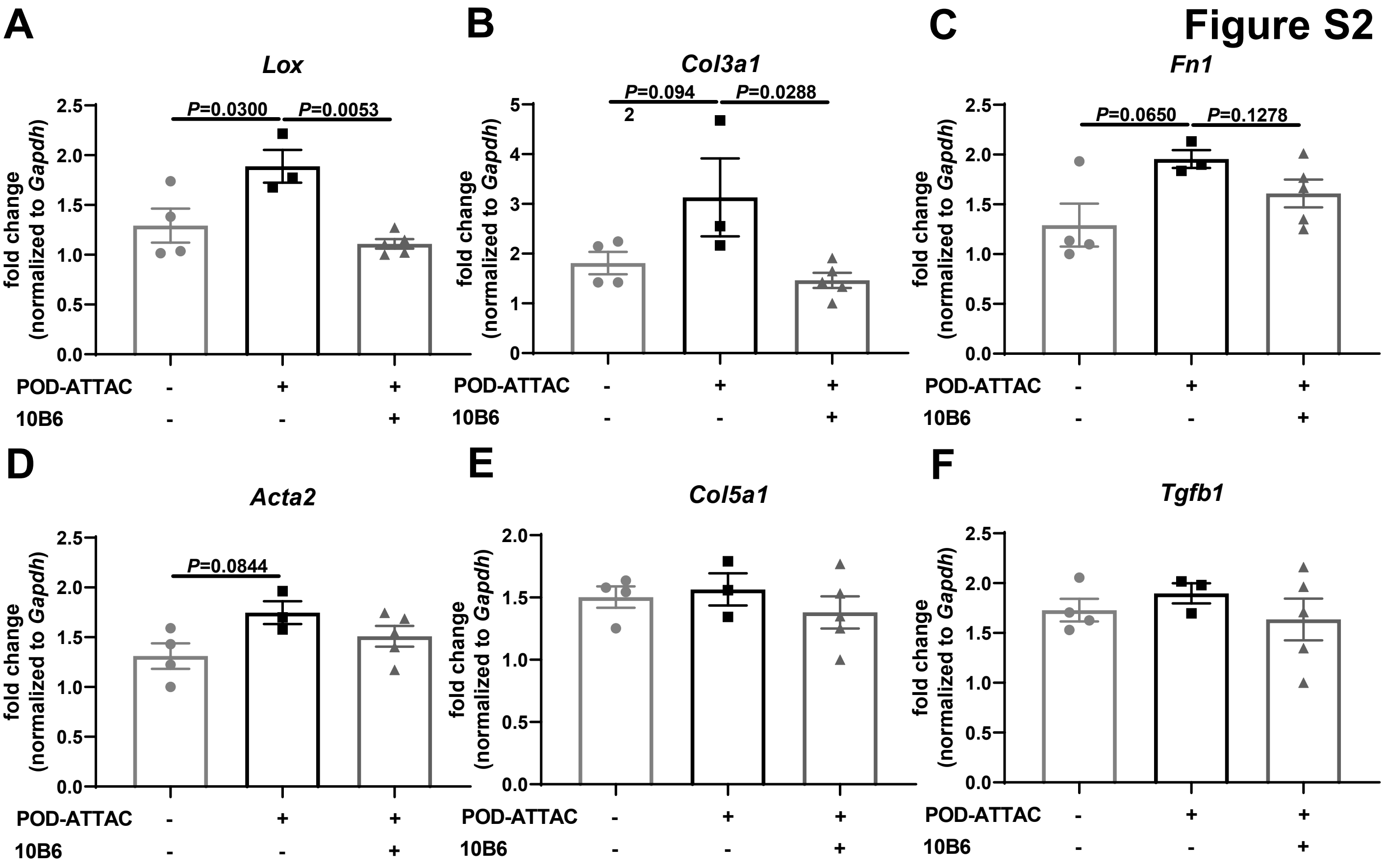

Fig. S2. Neutralization of endotrophin by 10B6 suppresses renal fibrotic gene programs. (A-F) Selective fibrosis related gene expression in kidneys from four groups: Lox, Col3a1, Fn1, Acta2, Col5a1, and Tgfb1. Data are presented as mean ± SEM of biologically independent samples. P values are shown. One-way ANOVA followed by a Tukey post-test (A-F).

Figure S3

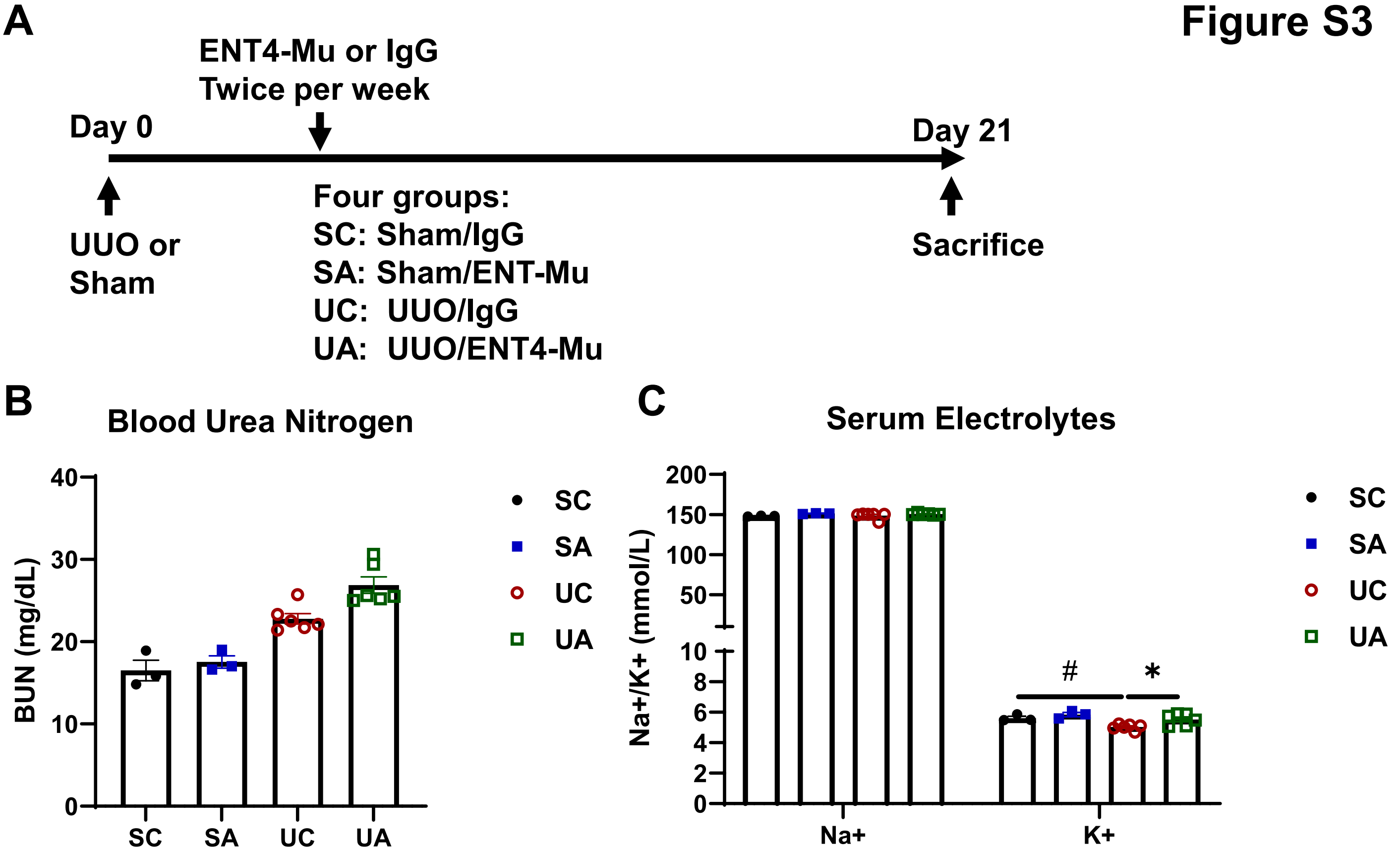

Fig. S3. Blockade of endotrophin by ENT4-Mu fails to improve renal function in the UUO model. (A) Schematic illustration of the treatment procedures. Sham or Unilateral Ureteral Obstruction (UUO) procedures were performed at Day 0, and then either control IgG or ENT4-Mu antibody (100µg/mouse) was given to either Sham or UUO mice. Four groups are shown here: SC, n=3; SA: n=3; UC, n=6; UA, n=6. (B-C) Blood urea nitrogen (BUN) (B), serum sodium (Na+) and serum potassium (K+) (C) levels in four groups. Data are presented as mean ± SEM of biologically independent samples. *P<0.05, #P<0.05. One-way ANOVA followed by a Tukey post-test (C).

Figure S4

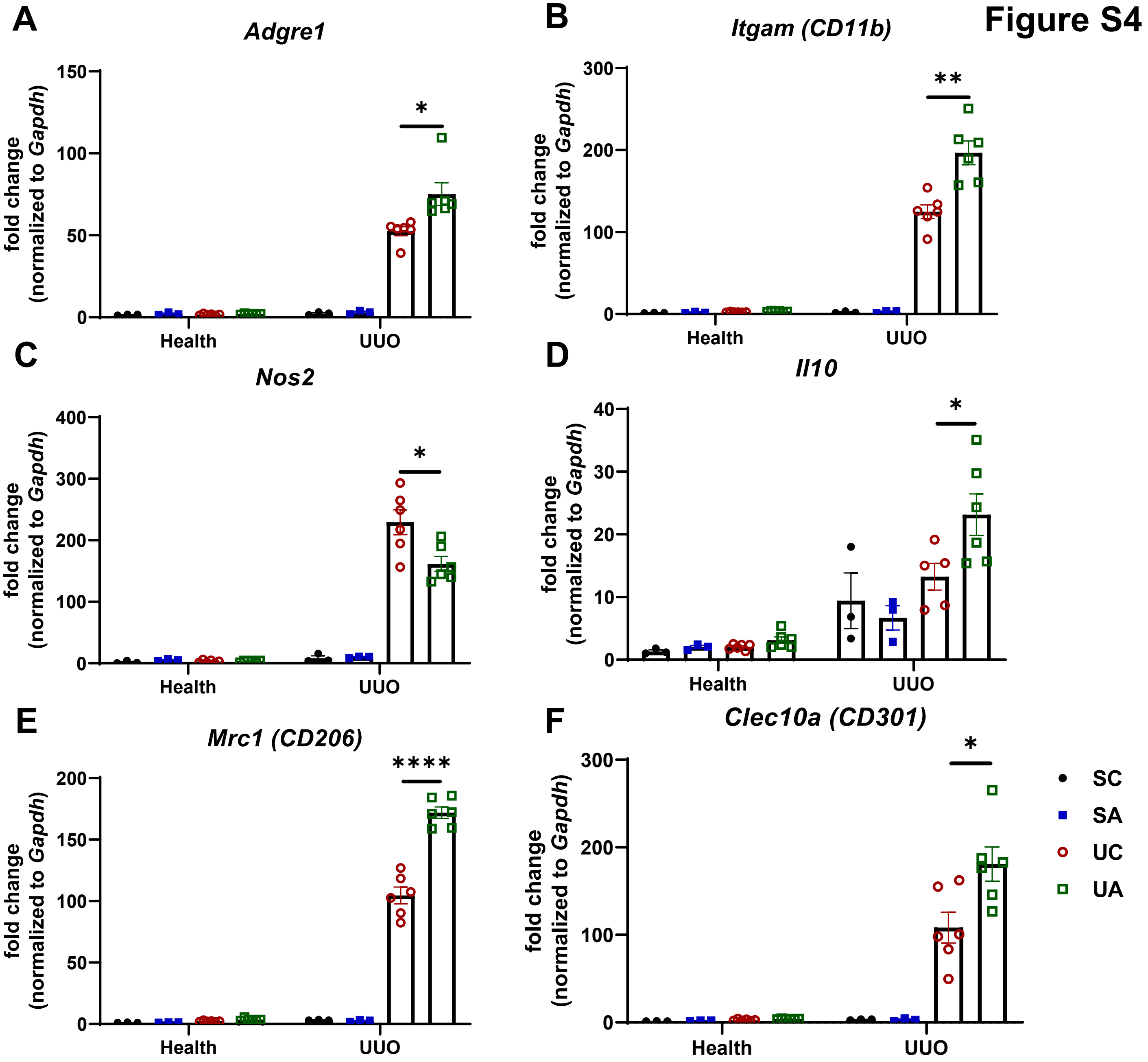

Fig. S4. Blockade of endotrophin by ENT4-Mu shows increased renal inflammation but promoted M2-like macrophage polarization in the UUO model. (A-F) Selective inflammation related gene expression in kidney macrophages: general macrophage markers, Adgre1 and Itgam; M1 markers, Nos2; M2 markers, Il10, Mrc1, and Clec10a. Data are presented as mean ± SEM of biologically independent samples. *P<0.05, **P<0.01, ****P<0.0001. One-way ANOVA followed by a Tukey post-test (A-F).

Figure S5

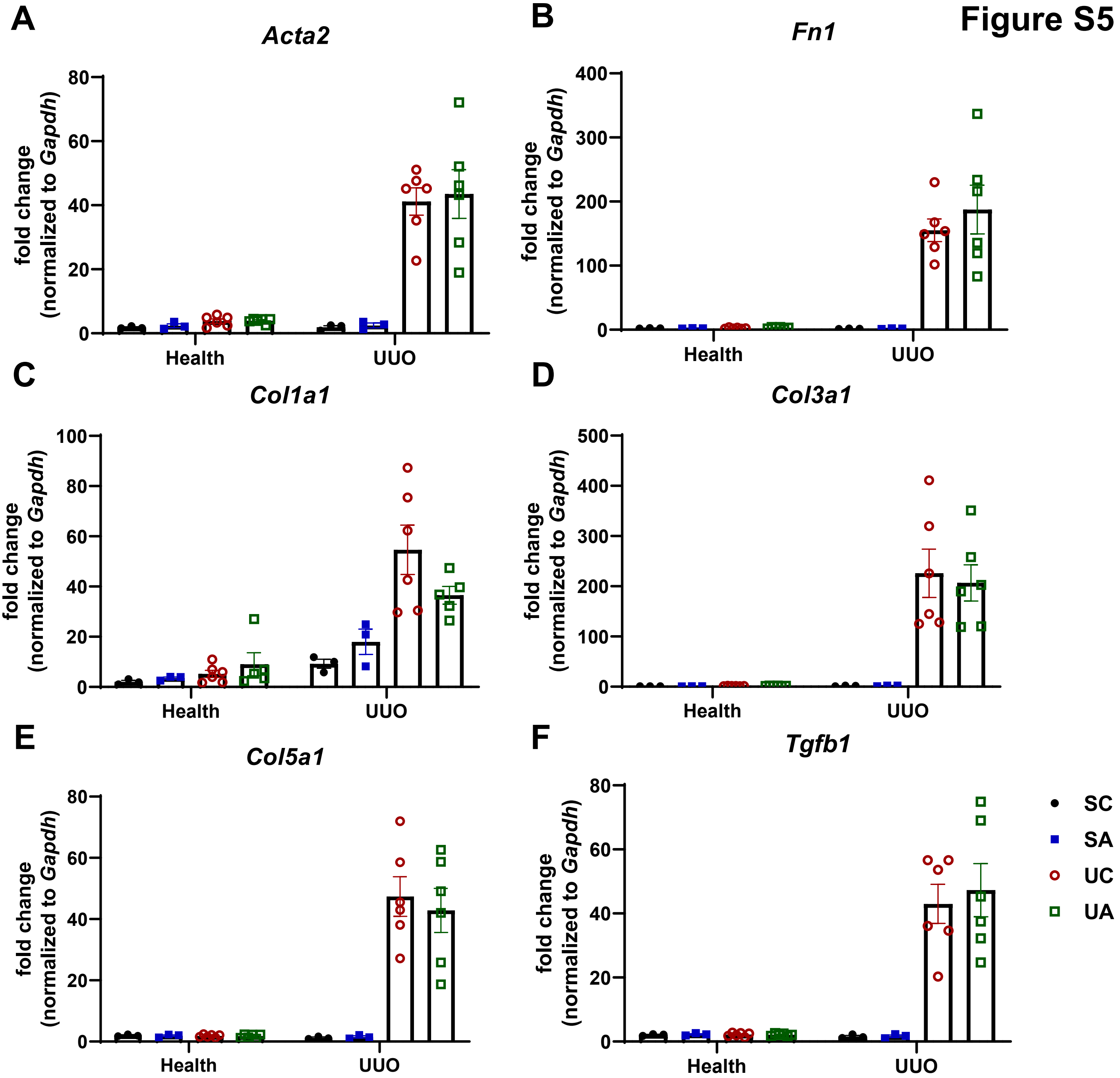

Fig. S5. Blockade of endotrophin by ENT4-Mu fails to suppress renal fibrotic gene programs in the UUO model. (A-F) Selective fibrosis related gene expression in kidneys from four groups: Acta2, Fn1, Col3a1, Col5a1, Col6a1, and Tgfb1. Data are presented as mean ± SEM of biologically independent samples. One-way ANOVA followed by a Tukey post-test (A-F).

Figure S6

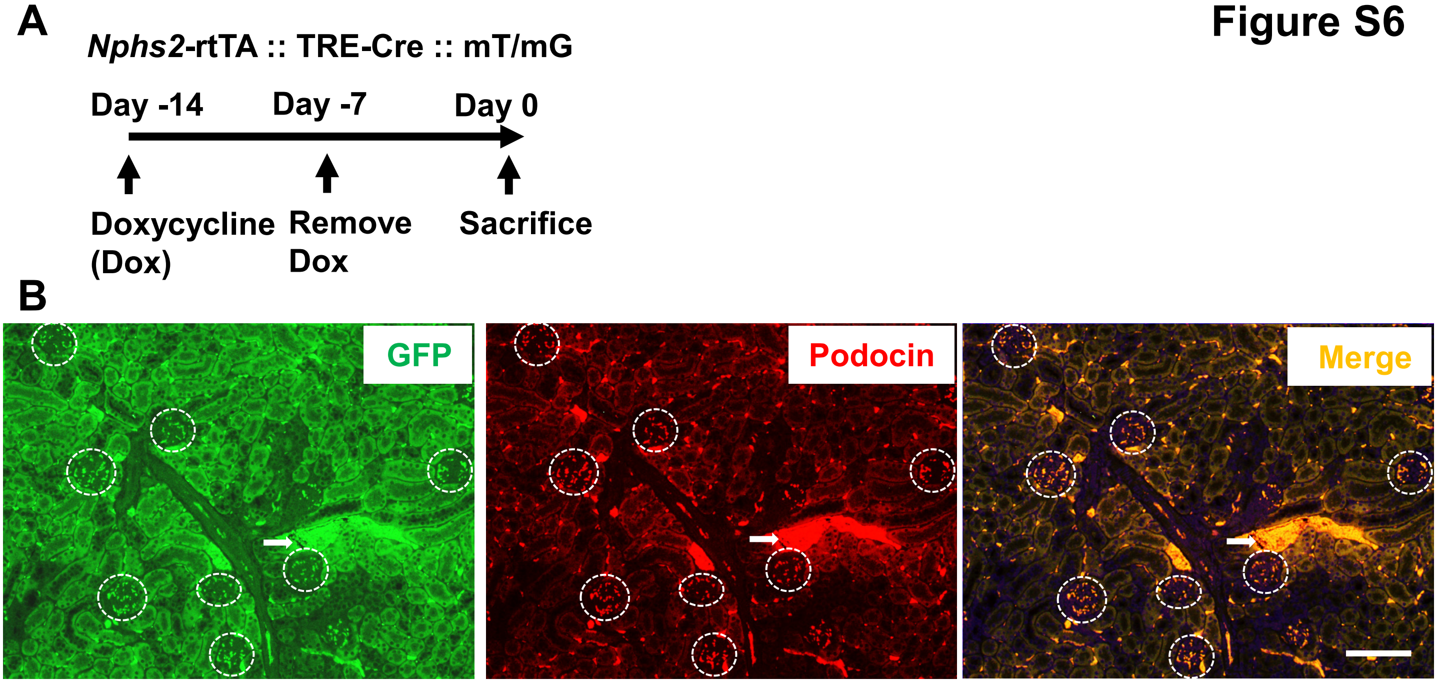

Fig. S6. Lineage tracing evidence supports high labeling of podocytes in "POD-Chaser" model. (A) Schematic illustration of the lineage tracing procedures. Doxycycline (Dox) was given to mice at Day -14 and removed at Day -7. After Dox washing out for 7 days, mouse kidneys were harvested at Day 0. (B) Kidney immunofluorescence staining of podocin, and GFP (podocin: red; GFP: green; merge: orange), bar = 161 μm. Circle indicates a glomerulus, and white arrow indicates unspecific staining of blood cells.
